# Programmatic implications for promotion of handwashing behavior in an internally displaced persons camp in North Kivu, Democratic Republic of Congo

**DOI:** 10.1186/s13031-019-0225-x

**Published:** 2019-11-20

**Authors:** Lauren S. Blum, Anicet Yemweni, Victoria Trinies, Mimi Kambere, Foyeke Tolani, Jelena V. Allen, Thomas Handzel, Susan Cookson, Pavani K. Ram

**Affiliations:** 1 Consultant, University at Buffalo, Buffalo, NY 14214 USA; 2 University of Kinshasa, Kinshasa, Democratic Republic of Congo; 3 University at Buffalo, Buffalo, NY 14214 USA; 4 OXFAM, Goma, Democratic Republic of Congo; 5 OXFAM, Oxford, UK; 6 Centers for Disease Prevention and Control, Atlanta, GA 30333 USA

**Keywords:** Handwashing behavior, Humanitarian emergencies, WASH, Hygiene promotion, Qualitative research, Democratic Republic of Congo

## Abstract

**Background:**

Diarrhea and acute respiratory infections (ARI) account for 30% of deaths among children displaced due to humanitarian emergencies. A wealth of evidence demonstrates that handwashing with soap prevents both diarrhea and ARI. While socially- and emotionally-driven factors are proven motivators to handwashing in non-emergency situations, little is known about determinants of handwashing behavior in emergency settings.

**Methods:**

We conducted a qualitative investigation from June to August 2015 in a camp for internally displaced persons with a population of 6360 in the war-torn eastern region of the Democratic Republic of Congo. We held key informant interviews with 9 non-governmental organizations and camp officials, in-depth interviews and rating exercises with 18 mothers of children < 5 years, and discussions with 4 groups of camp residents and hygiene promoters to identify motivators and barriers to handwashing.

**Results:**

At the time of the study, hygiene promotion activities lacked adequate resources, cultural acceptability, innovation, and adaptation for sustained behavioral change. Lack of ongoing provision of hygiene materials was a major barrier to handwashing behavior. When hygiene materials were available, camp residents reported that the primary motivator to handwashing was to prevent illness, particularly diarrheal disease, with many mentioning an increased need to wash hands during diarrhea outbreaks. Emotionally- and socially-related motivators such as “maintaining a good image” and social pressure to follow recommended camp hygiene practices were also reported to motivate handwashing with soap. Residents who engaged in day labor outside the camp had limited exposure to hygiene messages and handwashing facilities. Interviewees indicated that the harsh living conditions forced residents to prioritize obtaining basic survival needs over good hygiene.

**Conclusions:**

Hygiene promotion in camp settings must involve preparedness of adequate resources and supplies and ongoing provision of hygiene materials so that vulnerable populations affected by emergencies can apply good hygiene behaviors for the duration of the camp’s existence. Compared to non-emergency contexts, illness-based messages may be more effective in emergency settings where disease poses a current and ongoing threat. However, failure to use emotive and social drivers that motivate handwashing may present missed opportunities to improve handwashing in camps.

## Background

Diarrhea and acute respiratory infections (ARI) account for about 30% of deaths among children under five years living in refugee camps [1]. In acute emergencies, diarrhea is estimated to cause up to 40% of child deaths [2]. A wealth of evidence supports the positive impact of handwashing promotion for prevention of both diarrhea and ARI in non-emergency settings [3–7]. Although over 168 million people are estimated to be affected by humanitarian emergencies worldwide [8], little information is published in the peer-reviewed literature describing handwashing practices among emergency-affected populations. One study showed that, in three long-standing refugee camps in Thailand, Ethiopia and Kenya, handwashing with soap accompanied 30% of critical events such as before eating or after cleaning a child’s bottom or cleaning up feces and 20% of post-latrine use events [9]. Another study carried out in three recently established refugee camps in South Sudan found that handwashing with soap accompanied 22% of all critical events, 44% of fecal contact events, and 13% of food-related events [10]. A dearth of information regarding handwashing behavior among emergency-affected populations may hinder development and use of relevant strategies to improve hand hygiene in humanitarian emergencies.

In the non-emergency context, socially- and emotionally-driven factors, such as nurture, disgust, cleanliness, and affiliation, are important motivators to individual handwashing behavior [11, 12], and promotion of psychological drivers of behavioral change has been shown to improve handwashing, as well as nutrition practices [13–16]. Experts on water, sanitation and hygiene (WASH) in emergencies have asserted that strategies using health or disease avoidance as drivers for handwashing promotion do not sufficiently motivate handwashing with soap during emergencies except in outbreak situations [17]. A key obstacle to effective handwashing promotion identified by WASH experts was the limited understanding by those involved in program design and implementation of motivators and barriers to handwashing with soap in emergency settings [17]. WASH agencies and researchers are starting to explore psychosocial motivators and barriers to handwashing for emergency-affected populations, which could enhance handwashing behavior in the acute and subsequent phases of emergencies. One approach that can enhance contextual relevance is to identify emic or local views on motivations to handwashing behavior.

The Integrated Behavioral Model for Water, Sanitation and Hygiene (IBM-WASH) employs three intersecting dimensions, including the contextual, psychosocial and technological dimensions, that operate on five levels (structural, community, household, individual and habitual) to influence WASH behaviors [18]. This conceptual framework facilitates the examination of the multifaceted, dynamic factors affecting WASH behaviors and how the complex interactions of determinants operating at different levels shape handwashing practices. Using the IBM-WASH framework, we examined the ways in which contextual factors (the setting, natural environment, access to enabling resources, mobility, gendered division of labor, socio-demographic characteristics), psychological factors (psychological determinants, social norms and social desirability, knowledge and perceived threat of illness, community cohesion), and technological factors (location, design and physical characteristics, and availability of the hardware) collectively influenced handwashing practices in an emergency setting.

In a camp of internally displaced persons (IDP) in the Democratic Republic of Congo (DRC), we conducted a qualitative investigation to identify motivators and barriers to handwashing from an emic perspective. We also aimed to examine hygiene promotion activities carried out in the camp, availability of handwashing technologies and materials and perceptions regarding their appropriateness, hygiene and handwashing knowledge and behaviors prior to entering the camp, and how the camp conditions and sociocultural environment affected handwashing behaviors. Findings were used to inform trials of handwashing interventions in the camp environment [19].

## Methods

### Study setting and population

The study was conducted from June to August 2015 in Camp Kishusha, an IDP camp in Rubaya, North Kivu Province, in the war-torn eastern region of the DRC. The camp is situated on steep mountainous terrain where rain falls from August to May. Residents entered Kishusha in three waves perpetuated by escalations in hostilities, starting in July 2012 through October 2013. The camp was densely populated with people from different ethnic groups who had formally been in conflict sharing neighborhoods and camp facilities. Languages spoken were Kinyarwanda, Kihunde, Kiswahili, and French.

At the time of the study, the camp hosted 1328 families and 6360 residents and was demarcated into 49 blocks, with 25–30 households per block. Families lived in small tarpaulin-made tents spaced a few feet apart. Each block was supposed to have banks of functioning pit latrines shared by multiple families, with communal handwashing stations next to latrines. There were 57 functional communal pit latrine banks (each consisting of 2–4 latrine doors) throughout the camp, resulting in an average of one latrine bank per 112 residents. Several water pumps were available in the camp. Since the inception of the camp, different non-governmental organizations (NGOs) had provided residents with foodstuffs and basic needs, including materials for handwashing such as bar soap and water receptacles. The camp had experienced periodic cholera epidemics with the most recent occurring about three months prior to our study.

### Study design and sampling

We employed complementary qualitative data collection methods as described below.

*Key informant interviews* included NGO representatives implementing WASH activities, camp residents participating in camp governing and hygiene committees, and hygiene promoters overseeing routine hygiene activities. Eligible NGOs involved those promoting hygiene, as well as funding or coordinating organizations working at regional or country levels on WASH. Potential categories of NGO respondents included hygiene promotion managers and senior WASH staff coordinating activities with extensive experience in camp settings. Initial informants were selected purposively, and we subsequently used snowball sampling to identify additional informants.

With key informants we examined hand hygiene practices in the camp, handwashing promotion strategies, monitoring and evaluation of handwashing promotion activities, and coordination of handwashing activities with other organizations working in emergency settings. Aspects of handwashing promotion that were examined included factors that guided the development and evolution of the handwashing behavior change strategy, selection, distribution and acceptability of the handwashing hardware, perceived comprehensibility and persuasiveness of the communication messages, and challenges related to the implementation of handwashing activities.

Using a freelisting approach, at the end of each key informant interview we asked informants to list motivators or factors that stimulate handwashing behaviors. Specifically, we explained that we were interested in learning what motivates camp residents to wash their hands, with research assistants presenting examples of potential handwashing motivators to ensure that informants understood the exercise. Research assistants introduced the topic by stating, ‘We know that people wash their hands for different reasons, such as to feel good, feel clean or get rid of a bad odor, can you tell us why people in the camp wash their hands?’ Once an initial list of motivators was generated, we used probing techniques to elicit additional motivational drivers of handwashing until the list was exhaustive. After lists were generated by all of the key informants, we assessed the frequency by which each motivator was listed. A final list of seventeen of the most frequently listed motivators to handwashing was combined with three emotional drivers including ‘disgust,’ ‘attract other people’ and ‘habit’ proven effective in non-emergency contexts [11, 14]. Subsequently, all 20 motivators were translated by multilingual local translators into Kiswahili and Kinyarwanda, the two most commonly spoken languages in the camp, and subsequently back translated into French.

*In-depth interviews* were conducted with female caregivers of young children under five years old to explore knowledge of handwashing, past and current handwashing practices, motivators and barriers to handwashing, and perceptions of the handwashing promotion strategy in the camp. We purposively targeted females due to their role as primary child caregivers and responsibilities related to food preparation and maintaining household hygiene. We selected four blocks geographically separated in the camp. Interviewers were requested to find a central location in the block and spin a bottle to identify a household where they inquired whether a caregiver meeting our criteria was available to participate. If the approached household did not have a person eligible or willing to participate, the interviewer moved to the closest household to the left. When a potential respondent was identified, verbal informed consent was obtained and an interview scheduled. After the interview, the researcher approached another household five households to the left. This process continued until five mother-respondents in each block were interviewed. We anticipated administering 20 in-depth interviews or until reaching data saturation.

After in-depth interviews, rating exercises to assess perceptions of handwashing motivators were conducted with the same women. The structured rating task has been used successfully in communities with high rates of illiteracy [20]. Prior to implementation, the exercise was pilot tested with mothers not included in the study to assess their understanding of the motivators and ability to partake in the exercise. Appropriate revisions were made based on the piloting exercise. Subsequently, caregiver-respondents in our study were presented index cards one-by-one with the different handwashing motivators identified during key informant interviews and psychosocial motivators proven effective in non-emergency contexts written on separate cards [11]. After each motivator was presented, mothers were asked whether they understood the motivator, with research assistants providing additional explanations when needed. Respondents were asked to rate motivators on handwashing behavior on a three-step, bipolar scale. We began by asking each mother to identify which of all the motivational drivers was the strongest, which was the weakest, and which fell in-between in terms of motivating them to wash their hands, with research assistants once again describing each motivator to respondents. Once an initial range of three motivators was established on the three-step scale, each motivator was presented one-by-one and mothers were asked whether it was strongly motivating, not motivating, or somewhere in between. This process continued until all of the motivators had been rated. After completing the exercise, we revisited the way each motivator had been rated to confirm that the respondent was satisfied and to assess whether she wanted to make any changes in her responses.

*Group discussions* were carried out with separate groups of female caregivers of children under five years of age, male heads of households, and camp residents overseeing hygiene promotion activities. Main topics explored included motivators and barriers to handwashing and perceptions of the handwashing promotion strategy in the camp. Another purpose was to share preliminary findings from the key informants and mother-respondents and elicit recommendations to improve behavioral change approaches. Participants in groups of female caregivers and male household heads were selected in the four blocks where in-depth interviews were conducted with assistance from camp hygiene committee members and promoters and block leaders. We purposively selected communicative inhabitants who were available during the day to participate, with the target of conducting six group discussions.

### Data collection procedures

Data collection was conducted by two experienced male Congolese qualitative researchers not previously affiliated with camp activities with assistance from a female international investigator. Both Congolese researchers held Master’s degrees and the international researcher had a doctoral degree. Prior to data collection, a three-day training was led by the international investigator to introduce the study protocol, methods, and ethical procedures and to test and modify the instruments.

Initial data collection involved key informant interviews, which were carried out by the two Congolese researchers and the international investigator in a workplace where privacy could be maintained. Key informant interviews lasted approximately 1 h and continued for the duration of the study. During early interviews, we used a guide to address topics related to handwashing promotion, obtained information on the physical layout and sociocultural composition of the camp, and generated lists of motivators to handwashing. Subsequent interviews focused on clarification and interpretation of information gathered through other research methods. An iterative process involving the review of completed interviews and additional questioning was carried out by the two qualitative researchers until no new information emerged.

Each researcher was assigned two blocks where they conducted in-depth interviews and group discussions. Using an interview guide developed by the research team, efforts were made to administer in-depth interviews in a private setting, either in the household or an outdoor location next to the household, with each interview limited to 1 h 15 min duration. Techniques were used to probe for additional information according to respondents’ responses. If the researcher was unable to address all interview topics, a follow-up session was scheduled.

Group discussions were comprised of 8–12 people with similar background characteristics and held in a relatively private space, such as a church or a school classroom, with sessions limited to 1 h 30 min. One of the researchers moderated the discussions and another researcher took notes to facilitate data transcription. A study guide developed by the study team reflecting research themes and preliminary results procured through key informant and in-depth interviews was used to lead discussions, with adjustments in questioning made based on the content of discussions.

Key informant interviews were conducted in French or respondent’s preferred local language, and in-depth interviews and group discussions were administered in Kiswahili or Kinyarwanda with assistance from an interpreter. Interviews and group discussions were audio-recorded; interviewers took handwritten notes that provided additional insights into the data.

### Data analysis

Audio-recorded interviews were transcribed in Microsoft Word. Separate coding systems were developed for key informant interviews, in-depth interviews, and group discussions. Coding categories were derived from initial research themes and questions and key concepts that emerged during data collection. Coding of interview transcripts was done by one of the researchers and the international investigator on ATLAS.ti, a text-organizing software [21]. Content analysis was used to identify trends of concepts in and across individual codes. Rating exercises were analyzed on Anthropac 4.983 software [22]. The combination of data and methodological triangulation facilitated data analysis across research methods (key informant interviews, in-depth interviews, rating exercises, group discussions) and across and between respondents [23, 24].

## Results

### Descripton of respondents

Key informants included four NGO representatives, with three informants overseeing implementation and monitoring of WASH activities in emergency camps and the fourth informant working on community outreach. Key informants responsible for managing WASH activities had worked in emergency settings on average for over 10 years, while the community outreach worker had three years’ experience. These key informants had spent an average of 9 months delivering WASH services in the Kishusha camp. Two of these key informants were based in the provincial capital 63 km by dirt road from the camp and three participated in the cluster or working group in charge of coordinating WASH activities. We also conducted key informant interviews with the presidents of the camp administrative and hygiene committees and three hygiene promoters. Table 1 presents information on the key informants and other respondents interviewed.

**Table 1:** Research respondents according to data collection methods

Research methods and types of respondents	Total N
Key informants	
- Coordinator of public health activities, NGO representative	1
- Coordinator of hygiene, NGO representative	1
- Coordinator of NGO activities in the Kishusha camp, NGO representative	1
- WASH community outreach worker, NGO representative	1
- President of the camp administrative unit	1
- Head of the camp hygiene committee	1
- Hygiene promoters	3
In-depth interviews	
- Female caregivers of children < 5 years of age	18
Focus group discussions	
- Mothers of young children (10 participants)	1
- Male heads of households (10 participants)	1
- Members of the governing hygiene committee (12 participants)	1
- Block representatives overseeing hygiene promotion activities (8 participants)	1

We carried out 20 in-depth interviews with child caregivers. Unfortunately, two interviews were not audio recorded properly and thus information from 18 interviews was analyzed. The average age of respondents was 29 years (see Table 2); most were Protestant and had no formal schooling. The majority were married, with an average of four children and six people living in their households. Most were occasionally engaged in wage labor involving transporting goods or artisanal mining, while their husbands pursued these jobs daily. All households had a radio, mattress and telephone, and a few owned domestic animals. The average duration of residence in the camp was 31 months.

**Table 2:** Social and economic background information of mothers participating in in-depth interviews

Variables	Respondents (N = 18)
Age of mother (years)	
- Average	29
- Range	16–43
Religion	
- Protestant	17
- Catholic	1
Years of formal education	
- None	14
- Range among those who attended school (2–8 years)	4
Marital status	
- Married	16
- Single	1
- Divorced	1
Average number of children	3.8
Occupation prior to residence in camp	
- Farmer	16
- Small commerce	1
- Housewife	1
Occupation in camp	
- Day labor (porter, artisanal mining)	13
- Small commerce	3
- None	2
Number of months spent in camp	
- Average	31
- Range	16–36
Occupation of husband before entering camp	
- Farming	14
- Small commerce	1
- Phone repair	1
- Student	1
- No response	1
Occupation of husband while living in camp	
- Day labor (porter, artisanal mining)	11
- Farming	3
- Small commerce	1
- Phone repair	1
- No work	2
Average household size	6
Household assets	
- Radio	18
- Mattress	18
- Phone	18
- Animals	5
- Bicycle	1
- Chair	1

Four group discussions were conducted, including one group each of female caregivers of young children, male household heads, camp hygiene committee members, and residents overseeing hygiene promotion.

### Health concerns in the village and camp setting

Mother-respondents reported headache, flu, diarrhea, malaria, fever and stomach problems as frequent health conditions in the village. Diarrhea was cited as the most common health affliction in the camp, especially among young children. Explanations for the high frequency of diarrhea related to the dirty, crowded conditions and poor diets in the camp. Other commonly mentioned conditions in the camp included cough, fever and measles in young children, and stomach problems affecting all camp residents.

### Water, sanitation and hygiene activities

Key informants provided a detailed account of the hygiene activities implemented by the NGO initially in charge of WASH after the formation of the camp (Table 3) Handwashing interventions included setting up communal handwashing stations and the provision of soapy water and promotion of handwashing with soapy water at the public stations. The NGO assisted with the formation of a hygiene committee and ensured that volunteer hygiene promoters were identified in each block. With NGO support, the hygiene committee was responsible for a variety of tasks, including ensuring that communal handwashing stations were equipped and functional, hygiene promoters were working in each block, handwashing promotion activities were conducted, and hygiene practices were monitored and fines instituted when residents disregarded regulations. In November 2014, the mandate of the NGO originally charged with WASH activities ended due to their own internal guidelines, and a second NGO was appointed to oversee hygiene activities. Around the same time, soapy water was replaced by ash as the cleansing agent at communal handwashing stations. Our key informants indicated that funding could not sustain ongoing provision of soapy water and hygiene committee members proposed ash provided by female residents as an acceptable replacement.

**Table 3:** WASH activities in the Kishusha camp provided by NGOs in charge of WASH and the hygiene committee

Timing	Responsible group	Activities
Late 2013-early 2014	NGO in charge of WASH activities	-Set up water systems, latrines and communal handwashing stations -Distributed latrine maintenance kits to hygiene promoters -Distributed hygiene hardware such as soap and water receptacles to camp residents -Provided soap and promoted handwashing with soapy water at public handwashing stations -Assisted with the formation of a hygiene committee and training of committee members -Ensured that volunteer hygiene promoters were identified to work in each block
Early 2014-November 2014	Hygiene committee with assistance from NGO in charge of WASH activities	-Ensured pumped water was available -Ensured that hygiene materials were routinely distributed -Ensured that latrines and communal handwashing stations were equipped and functional -Made certain that hygiene promoters were working in each block -Ensured hygiene and handwashing promotion activities were conducted -Monitored hygiene practices and instituted fines when residents disregarded regulations -Involved in prevention and control efforts during cholera outbreaks (ensure chlorination of water sources, encourage boiling of drinking water, increase promotion of handwashing at critical times)
November 2014–June 2015	Replacement NGO in charge of WASH activities and hygiene committee	-Provided ash and promoted handwashing with ash at communal handwashing stations -Made sure hygiene promoters were working in each block -Decreased hygiene and handwashing promotion -Discontinued distribution of hygiene materials -Failed to ensure that latrines and communal handwashing stations were equipped and functional -Involved in prevention and control efforts during outbreaks -Instituted fines when residents disregarded regulations

At the time of the study, the hygiene committee was comprised of the original 12 members who continued to oversee WASH activities. Ongoing challenges mentioned by key informants included that there was only one water source, causing water shortages and permitting camp residents to receive only 5 liters daily, although some more distant water sources were available. The WASH community extension worker said,

*There is one water source that is shared by camp residents and the local community. The water is not sufficient. The water source is located on the farm of an army general. There are times when the farm manager closes the water and the camp residents suffer.*

The coordinator of NGO activities stated,

*In Kishusha, water is not sufficient. Only five liters of water is available per day per person. There is a need to increase the flow of water. Moreover, the same water source is shared with the host population.*

Key informants indicated that imited time periods and budgets awarded to NGO contracts restricted their ability to implement sustainable WASH activities and the longevity of the camp was uncertain, with the landlord threatening to force residents to evacuate. The NGO coordinator explained,

*The space occupied by the displaced is owned by an individual who is threatening to close the camp. . . The owner already started selling plots in the camp. New owners of the sold plots are threatening the IDPs because they want to start building.*

With respect to handwashing promotion, key informants contended that residents’ belief systems often conflicted with biomedically sound hygiene approaches, and time and budget constraints impeded broad-based behavioral change. The community extension worker said,

*I can say that their mentality, when we encourage them to wash their hands, they often say, ‘What is the foreigner coming to tell us? For a long time we survived without washing our hands and we did not get sick. . .’ They often follow their beliefs. They say that cholera comes from the air, from a bad wind.*

Later he added,

*Behavioral change is a process that cannot occur immediately. Strategies were introduced, but they did not always work. When you try to change the behavior of the population, 1 year is insufficient. If we can get funding for three or 4 years we will see real change. But if you are there for only one year it is not time to motivate action.*

We also learned that security restrictions prevented the donor agency from visiting the camp and visits by WASH cluster coordinators were rare.

During an initial walk-through of the camp, multiple signs suggested that hygiene activities were not receiving adequate attention. Several latrines were not functional, open defecation was apparent, and functional handwashing stations with ash were present at 20 (35%) of the latrine banks. Throughout the research period, we observed several potential hazards, including non-functional latrines which were not properly closed, and deep holes that had been created for latrines but were never used, which discussion group participants claimed had caused the demise of several residents who had fallen into the holes.

#### Hygiene promotion activities

NGO key informants reported that block presidents and hygiene promoters were oriented on good hygiene and handwashing practices and sensitization activities were initiated shortly after the first wave of IDPs entered the camp. All three hygiene promoter key informants reported never receiving formal training on hygiene, but rather were only given work directives from hygiene committee members. Representatives from the lead WASH NGO reported conducting group discussions to assess baseline hygiene knowledge and behaviors and adapt handwashing messages to coincide with residents’ understandings. Mother-respondents stated that during the early phase, NGO representatives promulgated information on what constitutes dirty substances, the importance of handwashing with soap to protect against germs and illness, critical times for handwashing, how best to wash hands with soap, and the dangers of exposing others to illness if soap is not used. The F diagram, which describes transmission pathways for fecal pathogens, and other health-related messages were primarily employed to promote handwashing practices [25]. Subsequently, handwashing messages were routinely disseminated by hygiene promoters and hygiene committee members during group meetings, household visits, or announcements on a megaphone; messages about hygiene and handwashing were also shared through theater skits and radio broadcasts. Hygiene promotion activities were reported to have intensified during a cholera outbreak in 2014 that spread to the camp population, but otherwise, message content remained the same.

In addition to awareness raising, two hygiene promoters stationed in each block were responsible for ensuring that water and a cleansing agent were available at communal handwashing stations, cleaning latrines and handwashing stations, and monitoring hygiene practices at communal stations. Hygiene promoters also conducted household visits to inspect for cleanliness and remind residents when and how to wash their hands. Work-related challenges mentioned by hygiene promoters at the time of the study included that the camp no longer provided soap for handwashing, the unwillingness of some inhabitants to subscribe to camp mandated hygiene and handwashing practices, and that hygiene maintenance equipment was old and not being replaced.

Mother-respondents lamented the decline in essential handwashing promotion activities, underlining that the NGO overseeing hygiene promotion at the time of the study discontinued provision of soap, communication activities had become sporadic or discontinued, and diarrhea once again posed a threat. One mother-respondent (P18) describing the decline in hygiene promotion commented,

*The diamond (previous handwashing promotion involving provision of soap and messaging about handwashing) has become a simple mineral (referring to the deterioration in handwashing promotion activities). . . Before it was good; now people are discouraged, awareness raising regarding good hygiene is not like before.*

Several mother-respondents suggested that reductions in food distribution and the discontinuation of soap had negatively impacted activities of the hygiene promoters and committee members, with one (P3) stating,

*Those overseeing hygiene have succumbed to a state of lethargy due to lack of motivation. They have become negligent in their work.*

Hygiene promoters and committee representatives continued to monitor hygiene practices and fine residents who did not adhere to camp rules.

#### Materials distributed

Key informants reported that initially hygiene kits including jerry cans, plastic buckets, brooms, rakes, squeegees, shovels, water taps and soap were distributed to hygiene promoters for latrine maintenance. At the time of the study, maintenance materials had not been distributed for over six months, and recently constructed latrines did not have handwashing stations. NGOs had also distributed basic household materials such as water containers, dishware and utensils, and a tarpaulin just after IDPs entered the camp, as well as a basin, jerry can, goblets, buckets, and bar soap for handwashing, which were generally appreciated by mother-respondents. One mother-respondent (P18) said,

*These materials are helpful, they are all we have, we are poor. Flight from our villages did not allow us to bring these items. . . I like these materials, it made me feel good when they gave it to me because I felt they were looking out for me. We had nothing when we arrived here.*

However, many mothers reported that hygiene materials had been stolen when they were outside the camp or they had not received hardware for over a year and what was previously distributed was old and no longer functional, or in the case of bar soap, had been used long ago.

#### Handwashing stations

Most mother-respondents valued communal handwashing stations, highlighting the convenience and fact that they serve as a reminder of handwashing after latrine use and enable hygiene promoters to monitor handwashing activities. They preferred removing “dirty substances” immediately after latrine use to avoid running the risk of having to greet people on the path home with unwashed hands or forgetting to wash their hands and contaminating others. Minimal space for handwashing at home and the desire not to deplete home water supplies for handwashing also favored communal stations. However, in one study block the communal station had stopped functioning  three months prior to our study, forcing residents to carry water to the latrine or to wash their hands in their homes or adjacent blocks, triggering conflict with block inhabitants. In another block, the latrines were no longer used because they were full, causing handwashing stations to become obsolete. Generally, mother-respondents and group discussion participants complained that communal station hardware was in poor condition or non-existent, and that since the NGO changeover, maintenance of stations was less rigorous, diminishing handwashing practices. One female group discussion participant said,

*Of the 12 hygiene committee members, there are only 2 who live in a block where stations are functional. You can ask them. They come and give us messages about practices they themselves cannot follow. Who can follow the messages they give us?*

Although mother-respondents living in blocks where stations were functional stated that water is generally available, ash was often not. Many maintained that only soap is effective in removing dirty and hard to remove substances like oil or dirt and eliminating “microbes” that cause illness, particularly diarrhea. Mother-respondents explained that washing without soap is equivalent to not washing, making them vulnerable to illness. Soap was also considered preferable due to its multi-faceted uses, including washing the body, clothes and dishes, as well as it being manufactured, which signified high quality and special ingredients. Many mothers indicated that only soap motivates handwashing.

Mother-respondents and group discussion participants reported that ash does not remove all dirty substances associated with illness or have cleansing capabilities, with many never previously using ash for handwashing. Other disadvantages cited were that ash leaves a white substance on the hands, does not make the hands feel good, makes handwashing hardware dirty and less attractive, is not valued, and is only used for handwashing after using the toilet, but not during other events or for other purposes. A male group discussion participant said,

*It is difficult to say that ash prevents illness the same way as soap, because ash does not have a chemical composition that guarantees the same effect. Ash is natural. It is impossible to wash clothes with ash. Only soap is effective in ridding clothes of dirt. This leads people to have more confidence in soap than ash.*

Group discussion participants reported a perceived increase in diarrhea and cholera since the discontinuation of soap.

### Consequences of other NGO Activities on Hygiene Practices

NGOs overseeing hygiene were also involved in food distribution. At the time of the study, NGO key informants reported that less than 25% of households met donor criteria related to food insecurity and camp inhabitants were encouraged to seek employment to provide for their families. Mother-respondents and group discussion participants cited inequities in receipt of food, which they reported affected morale, caused conflict, decreased NGO credibility, and served as a disincentive to subscribe to good handwashing practices. One female group discussion participant said,

*When NGOs distribute food in the camp, they give to some but not others. When our neighbor does not eat, he will not be in good health. Health is not only about providing water and handwashing.*

Several mother-respondents indicated that feeding family members is of greatest concern, and without sufficient food, hygiene and handwashing initiatives lose importance.

### Handwashing Knowledge and Behaviors

#### Prior to arriving in the camp

Study participants indicated that their natal villages were in isolated areas where hygiene information was limited or non-existent, with some suggesting that the sparse populations rendered handwashing unnecessary. One hygiene committee member said,

*I could eat without washing my hands because people in the village were few, but here in the camp there are many people. For me many people bring uncleanliness. That is why I started to wash my hands.*

Mother-respondents reported using only water for handwashing, explaining that soap was unaffordable and they had been unaware that ash cleansed. Most indicated that family members washed their hands in a common receptacle, with men sometimes provided a separate basin. Mentioned times for handwashing included after waking from sleep, after farming, before eating and when they bathed. One mother-respondent (P2) said,

*Because I lived in a secluded area, I had no idea about hygiene. After farming, it was sufficient to dip our hands in water and then we ate. We put the water in a receptacle and we washed with it until it got dirty. Then we threw it away.*

Several mothers maintained that it was important to wash hands before church or when receiving guests. Other barriers to handwashing included distances and time required to obtain water and the need to eat quickly when sharing a communal plate with family members.

#### While residing in the camp

Mother-respondents reported that initially residents ridiculed handwashing messages because they were unaware of the importance of hand hygiene. However, they claimed that exposure to the same communications from different sources and experiences involving residents either complying or not complying with handwashing promotion strategies in the camp where disease was perceived as a persistent threat, confirmed their veracity and led to the general acceptance by mothers of promotional messages. These respondents consistently maintained that following good handwashing practices protects their health, particularly against diarrheal disease. Several made reference to a diarrhea outbreak which killed camp residents, raising fear and validating that handwashing saves lives. One mother-respondent (P5) stated,

*We were taught the rules of hygiene and the number of illnesses diminished. If we cannot follow these rules, it is due to our habits from the village. We can see that following the handwashing messages has helped us. If we do not, we pay the consequences (by getting sick).*

Pouring water over the hands was described as the best handwashing approach. Reported critical times to wash hands included before food preparation or consumption, before breastfeeding, after household chores or work outside the camp, when the hands are visibly dirty, and after getting up from bed, with mother-respondents stressing heightened risk of contamination after shaking hands, using the latrine or washing a child who has defecated. A mother (P1) stated,

*When we walk around the camp we greet many people, we don’t know what they have touched. When we arrive home, we must wash our hands.*

All mothers reported that failure to wash hands before food preparation and consumption can lead to ingestion of dirty substances causing diarrhea, vomiting or other illness, with some mentioning exposing family members who eat from the same receptacle. Mother-respondent (P1) claimed,

*Illness is caused by contact, and if you have eaten without washing your hands, you have eaten dirty substances or microbes that leads to illness.*

Most mother-respondents reported washing their hands more often than other household members due to their involvement in food preparation, exposure to dirty items while cleaning the household, caring for animals and providing childcare, and ongoing access to water. The majority claimed to remind others, particularly children, to wash hands. This mother (P16) said,

*Here, if someone is exposed to germs it is a danger for everybody. That is why it is important to remind others to wash their hands.*

At the time of the study, mothers reported washing their hands at public stations or at home. Many indicated that handwashing had become a habit when soap was available, but the majority refused to use ash. One mother-respondent (P13) said,

*Now it is ash, since the departure of X (previous NGO), soap is not available. Maybe it is for this reason many people are no longer interested in washing their hands each time (they use the latrine). Lack of soap discourages people from washing their hands.*

A hygiene committee member said,

*When people leave the toilet, they don’t find soap by the handwashing station, they only see ash and say, ‘I will wash at home.’ There is the risk of forgetting.*

Most respondents reported handwashing with plain water, which they recognized to be ineffective in removing dirty substances.

### Motivators and Barriers to Handwashing Behavior

#### Motivators

The most commonly cited motivator for handwashing by mother-respondents was to rid the hands of dirty substances or “microbes” and thus protect against illness, with diarrhea mentioned most frequently. The camp was described as a very dirty place, raising fears about contracting illness and necessitating that residents wash their hands to maintain health, especially during diarrhea outbreaks, with some underscoring that as caregivers they must stay healthy. Limiting illness transmission to family members, particularly young children who they considered highly susceptible, was also mentioned as a key motivator. Results from the rating exercise presented in Table 4 confirms that handwashing is primarily driven by illness-related attributes, with “protect children from illness,” “rid the body of germs,” and “avoid illness” considered most compelling.

**Table 4:** Rating results of handwashing motivators

Ranking	Motivator in English	Mean Score*
1	Protect children from illness	1.17
2	Rid the body of germs	1.39
3	Avoid illness	1.44
4	Clean	1.50
5	Fear of illness	1.56
5	Get rid of bad odor	1.56
7	Good health	1.61
8	Good example	1.72
9	Avoid contaminating others	1.78
9	Disgust	1.78
11	Feel good	1.83
11	Smooth, soft	1.83
13	Joy	1.89
14	Avoid embarrassment	1.94
15	Attract other people	2.00
15	Gives self-assurance, confidence	2.00
15	Wash the body	2.00
18	Habit	2.06
19	Pride	2.44
20	Social pressure	2.50

*The mean scores were generated on Anthropac using 1 for the best motivators, 2 for moderately good motivators, and 3 for least effective motivators

Soap was reported to encourage handwashing, with some asserting that the lather eliminates dirty substances and conveys cleanliness. Other attributes included that soap makes hands smell good, feel light, smooth, and soft, and look clean and pretty, making the user feel good, at ease and confident. Some mothers suggested that clean hands engender pride and boosts self-esteem, and eliminating offensive odors that cause embarrassment enhances their image (Table 5). Maintaining a good image was considered particularly important during Sunday church. Many respondents alluded to social pressure to conform to camp hygiene rules including handwashing.

**Table 5:** Motivators of handwashing behavior, as reported by study respondents

*When you use soap you smell good, the hands are smooth, and when you are with others, you feel you smell good and are not concerned about emitting a bad odor.* (In-depth interview mother-respondent P18) *Before going to church, people must wash their hands with soap so that the hands are pretty and clean and they feel comfortable shaking hands and greeting others.* (In-depth interview mother-respondent P13) *We feel happy, this is the sentiment you feel washing the hands (with soap) after using the latrine, the feeling of joy and pride. It gives us a peace of mind and we do not feel guilty, you will not have any concern about infecting someone else when shaking their hand on the road.* (Male group discussion participant) *There are two things we are told. If you decide to settle in the camp, you have to follow the hygiene rules. If you don’t, you risk contaminating others. If you do not follow the rules, you must return to the village.* (In-depth interview mother-respondent P5) *Here in the camp, we have had to make many adjustments. First, we have no work, we remain idle. The instructions we receive do not permit us to eat without washing our hands, which we follow out of fear that the community is going to condemn or make fun of us.* (Male group discussion participant)	

#### Barriers

The most common reason for failing to wash hands related to preoccupations with other pressing issues, particularly that family members eat sufficiently (Table 6). Some mothers maintained that during mealtimes, family members rush to eat and may forget handwashing. Lack of visible dirt on the hands was also reported to diminish the perceived need for handwashing.

**Table 6:** Barriers of handwashing behavior, as reported by study respondents

*I lose my mind when my children don’t eat at night and I don’t know how we are going to eat. I start wondering how the children will survive, and it isn’t possible to think about handwashing. . .*. *Because we lack food occasionally, we do not always follow all of the (hygiene) rules. Those in charge of hygiene work well, but people cannot think clearly when they don’t have anything to eat.* (In-depth interview mother-respondent P19) *If I see that my hands are dirty, I wash my hands because I have greeted many people on the path. But if my hands do not look dirty, I do not wash my hands.* (In-depth interview mother-respondent P15) *I don’t know why. I see many people entering the toilet, do their business and leave without even washing their hands. It is just their bad habits. Really, someone who leaves the toilet without washing his hands, does he follow good hygiene at home?* (In-depth interview mother-respondent P13)	

Other explanations for neglecting to wash hands included that people working outside the camp are too busy, handwashing stations are non-functional, soap is no longer provided in the camp and unaffordable, or some people refuse to modify habits. Group discussion participants mentioned that people engaged in wage labor, who are mostly men, have less exposure to handwashing hardware and camp-based messaging, while female residents have greater contact with hygiene promoters.

## Discussion

In the DRC camp, hygiene promotion was introduced shortly after the camp was formed. However, study results show that two years after initiation of WASH activities, hygiene promotion was hampered by inadequate resources, and lack of cultural acceptability, innovation, and adaptation for sustained behavioral change. Camp residents reported that handwashing was primarily motivated by the desire to protect against illness, particularly diarrhea disease, reflecting camp-based messaging which appeared to garner acceptance in the congested camp where ongoing disease threats prevailed. Emotionally- and socially-related motivators were also reported to stimulate handwashing with soap. The change from providing soap to promoting ash and failure to replace old handwashing technology prevented residents from handwashing with highly valued soap and made them opt to wash with plain water. Results give special insights into the challenges of hygiene promotion in an IDP camp where harsh living conditions and rampant poverty forced residents to prioritize basic survival needs over following good hand hygiene behavior.

We found that communication strategies lacked clear objectives, innovation and a long-term vision, thus undermining sustained and widespread behavioral change. Hygiene knowledge of study participants mirrored a hybrid of cultural beliefs and customs held prior to entering the camp and knowledge and practices influenced by the camp context, hygiene messages and rules. A prevailing concept supported by study respondents was that handwashing with soap is effective in protecting against illness, reflecting the disease transmission and prevention focus of camp messages. Although NGOs reported conducting formative research, there was no indication that hygiene sensitization was adapted to residents’ beliefs and practices or the evolving camp context. Rather, the same standard messages were disseminated from the camp inception, with modifications occurring only during diarrhea outbreaks. Still, the ongoing perceived threat of diarrhea disease in the densely populated camp environment appears to have influenced acceptability of illness-based messages. One theory is that when disease symptoms are clear-cut, the disease threat is pervasive, and effective measures are available, cause and effect problem solving is heightened [26]. In this instance, firsthand experiences with diarrheal illness, particularly during diarrhea disease outbreaks in the camp setting, may have convinced IDPs about the veracity of germ and disease transmission theory and the concept that handwashing with soap protects against diarrhea. Notably, study participants never mentioned prevention of ARI, which accounts for a large proportion of child deaths during humanitarian emergencies [1]. This may be related to cultural perceptions associated with ARI compared to diarrhea, which has distinct, easily recognizable signs and symptoms, as well as to hygiene promotional activities that focused on diarrheal illnesses. Contact events related to exposure to respiratory illness should also be promoted as a critical time for handwashing in emergency settings.

While motivators to handwashing were primarily related to illness prevention, residents cited other emotionally- and socially-related motivators such as “feeling clean” or “being proud” and “maintaining a good image” that stimulated handwashing with soap. The mix of research methods provided a rich combination of data which allowed us to triangulate and validate the results related to handwashing motivators. Findings show that it was particularly important for mother-respondents to maintain a clean appearance and fresh smelling hands during public events such as Sunday church, one of the rare opportunities women had to socialize and project their status in the community. In the DRC, an emphasis to look good prevails, particularly on Sunday, when community interactions are heightened and Congolese try to convey an image of well-being and prosperity, which involves being clean, smelling good and dressing well (personal communication). Our respondents also unscored social pressure to follow camp hygiene regulations designed to diminish disease transmission to fellow residents. Social science literature has illuminated the importance of social rather than just individual change, since the former better ensures widespread acceptability and long-term impact of healthy behaviors [19]. A socially based approach promoting handwashing to protect the health of residents and to meet social standards of cleanliness and appearance may be particularly appropriate in a densely populated camp setting where people live in proximity, risk perceptions of disease transmission may be heightened and pressure to conform to social norms is pervasive.

Study results revealed the failure to maintain latrines and communal handwashing stations and distribute materials needed to apply good hand hygiene practices. While residents believed in the effectiveness of soap in preventing illness, they were unable to continue handwashing with soap due to the poor state of the public handwashing stations, discontinuation of soap distribution, and fact that soap was unaffordable to purchase. A quantitative study showed handwashing at communal stations to be far less common when residents were provided ash compared to when they were provided soap [19]. Ironically, NGOs implementing handwashing promotion did not investigate whether ash would be acceptable, with the decision driven by funding constraints. Residents maintained that ash was not previously known as an effective cleanser nor did it evoke health or socially- or emotionally-driven attributes that motivated handwashing. Alternatively, residents opted to wash with plain water, which was generally available at the communal stations. The preference to wash hands at public stations was partially influenced by a desire to economize the limited water supply allotted to families. At the time of the study, camp residents were receiving 5 liters of water daily, which is far less than the 15 liters recommended according to SPHERE standards [27].

The transfer to ash also coincided with the changeover from one highly respected NGO to an organization which reportedly did minimal work, leading to a sharp decline in camp hygiene activities. While the hygiene committee continued functioning, their work seemed in part to be galvanized by the desire to collect fines instituted when residents failed to follow camp rules. Study results point to inadequate oversight and commitment by NGO implementers and the WASH cluster regarding maintenance of effective hygiene promotion activities and a safe living environment. NGO representatives providing assistance often lived far from the camp, permitting only sporadic visits, which likely did not allow them to appreciate the intricacies of camp life and residents’ needs. A camp setting like Kishusha, where inhabitants representing different ethnic backgrounds had previously been in conflict, can be complex, thus requiring a regular presence to understand the social dynamics needed to develop effective handwashing approaches. In addition, security restrictions prevented donors from making firsthand situational assessments and informed decisions about funding needs. The short funding mandates allotted by donors and internal NGO policies restricting time commitments in a camp setting appeared to limit NGO capacity to implement relevant and sustainable strategies. Findings also suggest that NGO implementers had limited understanding of effective handwashing communication approaches and behavioral change theory. WASH experts have reported that inadequate expertise, failure to contextualize communication approaches, and lack of a formal behavioral change strategy undermines the success of hygiene promotion in camp settings [17]. These findings reinforce the need to involve behavioral change experts in the development and implementation of handwashing strategies in humanitarian emergencies and to inform NGO representatives working in remote locations about successful new and innovative social and behavioral change approaches.

Results highlight how the precarious nature of a camp setting can impede handwashing promotion activities, illustrating that basic survival needs take precedence, rendering hygiene practices secondary. NGO-led food distribution targeted a small percentage of families considered food insecure, causing resentment and potentially affecting willingness to adhere to other NGO recommendations regarding hygiene and handwashing practices. IDPs were expected to seek wage labor to eke out enough money to feed family members. When away from their homes, they fell victim to crime, including stealing of hygiene hardware. Those working outside the camp constituted a hard to reach group whose absence during the day limited exposure to handwashing messages and technology. Handwashing promotion should take into consideration the daily patterns of camp residents and modify approaches to better ensure all residents are reached through strategies.

### Study limitations

A limitation to this study was that we only carried out in-depth interviews with women. While group discussions included male household heads, because discussions were held during the day time we did not include males who routinely work outside of the camp. Failure to collect information from hard to reach males regarding their exposure to and perceptions of the handwashing promotion activities and motivators and barriers to handwashing behavior was a missed opportunity. Another limitation is that some respondents were at times reticent to openly share information. We suspect this relates to the history of camp residents, who had fled conflict zones prior to entering the camp, and historical ethnic tensions of groups residing in the camp, raising suspicion amongst residents. While the researchers were highly skilled, they were not from the study area and therefore did not speak the local languages.

## Conclusions

Motivating and sustaining optimal hand hygiene practices among emergency-affected populations is multifaceted and requires a long-term vision and strategy in prolonged emergency situations. In Kishusha, assistance given during the emergency phase appeared to be extensive and well received, but over time a deficit in hygiene promotion resources prevented residents from applying the handwashing knowledge acquired and practices encouraged in the camp where disease threats persisted.

Illness-based messages may be more effective in camp settings where overcrowding and suboptimal WASH conditions heighten the risk of infectious disease transmission compared to non-emergency contexts, likely altering perceptions of disease threats. However, lack of exploration and use of emotive and social drivers that stimulate handwashing may present missed opportunities to incorporate approaches proven effective in improving handwashing in development settings. The findings underscore the need to design contextually relevant approaches that follow sound hygiene practices and to modify handwashing strategies as camp conditions and resident’s knowledge base change. The poor state of WASH and other services uncovered during the study emphasize the need to provide acceptable and quality assistance that meets basic standards for the duration of the camp’s existence. Additional research to assess whether health-related or psychosocial motivators are more effective can help to prioritize messages related to hygiene promotion during emergencies.

## Data Availability

The datasets used and/or analyzed during the current study are available from the corresponding author on reasonable request.
